# RESEARCH ADVANCES USING DIGITAL METHODS TO ASSESS COGNITION IN OLDER ADULTS

**DOI:** 10.1093/geroni/igae098.2210

**Published:** 2024-12-31

**Authors:** Karysa Britton, Zoe Hawks, Martin Sliwinski

**Affiliations:** Chair; Co-Chair; Discussant

## Abstract

This symposium highlights the use of digital methods to assess and contextualize cognition in older adults. Across five talks, we consider several digital cognitive assessment methods, including self-administered computerized tests and smartphone-based ecological momentary assessments (EMAs). First, Dr. Louisa Thompson reports findings on the utility and acceptability of using well-validated, reliable digital cognitive assessments to screen for cognitive decline among older adults in primary care settings. Second, Dr. Zoë Hawks uses EMA and passive data from wearable sensors to investigate links between glucose and cognition in relation to neuropsychiatric risk. Third, Hannah Wilks discusses the importance of assessing both environmental and affective influences when examining cognition via EMA in individuals with symptoms of dementia. Fourth, Dr. Karina Van Bogart uses multilevel modeling to examine associations between levels of loneliness and self-reported memory lapses among older adults. Finally, Dr. Emorie Beck examines psychosocial influences on diurnal cycles of cognitive variation. Together, Dr. Martin Sliwinski will draw connections between research showcasing how digital methods can be employed to promote routine screening for cognitive decline, elucidate contextual factors that contribute to cognitive variation in older adults, and assess unique influences of individual differences on cognitive performance.

